# Effects of Low Intensity Focused Ultrasound Stimulation Combined With Functional Electrical Stimulation on Corticospinal Excitability and Upper Extremity Fine Motor Function

**DOI:** 10.1002/brb3.70318

**Published:** 2025-02-11

**Authors:** Naaz Desai, Talyta Grippe, Tarun Arora, Amitabh Bhattacharya, Carolyn Gunraj, Robert Chen

**Affiliations:** ^1^ Krembil Brain Institute University Health Network Toronto Ontario Canada; ^2^ Dept of Physical therapy University of Toronto Toronto Canada; ^3^ Edmond J. Safra Program in Parkinson's Disease, Morton and Gloria Shulman Movement Disorders Clinic, Toronto Western Hospital University Health Network Toronto Ontario Canada; ^4^ Division of Neurology, Department of Medicine University of Toronto Toronto Canada; ^5^ Division of Clinical Neuroscience, Department of Neurology Oslo University Hospital Oslo Norway; ^6^ Institute of Medical Science University of Toronto Toronto Canada

**Keywords:** functional electrical stimulation, motor cortex, plasticity, transcranial focused ultrasound

## Abstract

**Introduction:**

Functional electrical stimulation (FES) is used to retrain motor function in neurological disorders but typically requires multiple sessions and shows limited benefits in chronic cases. Low‐intensity transcranial focused ultrasound stimulation (TUS) is a noninvasive brain stimulation (NIBS) method offering greater focality and deeper penetration than current NIBS techniques. TUS delivered in a theta burst pattern (tbTUS) for 80 s produces neuroplastic changes with long‐term potentiation‐like effects lasting up to 60 min in healthy adults. Since tbTUS increases cortical excitability, combining it with FES may enhance neuroplasticity. We hypothesized that combining tbTUS with FES would result in increased corticospinal excitability compared to FES alone and lead to greater improvement in fine motor skills as assessed by Nine‐Hole Peg Test (NHPT) scores.

**Methods:**

Fifteen healthy participants underwent two study visits consisting of real or sham tbTUS of the left motor cortex immediately followed by 30 min of FES of the first dorsal interosseous (FDI) and the opponens pollicis (OP) muscles for fine motor function training of the right hand. Motor‐evoked potentials (MEPs) were recorded from the right FDI, OP, and abductor digiti minimi (ADM) muscles at baseline (BL), immediately after real or sham tbTUS (T0), immediately after 30 min of FES training (T45), and at 15 (T65) and 30 min (T80) post‐FES. NHPT was delivered at BL and at T80.

**Results:**

Data from 14 participants were analyzed. It showed a significant decrease in MEP amplitudes of FDI and OP at T45 following only real tbTUS+FES with a return to BL at T80. No significant changes were seen in the NHPT scores in either condition.

**Conclusion:**

Real tbTUS+FES combined with voluntary movement results in immediate corticospinal inhibition with a return to BL at ∼20 min post‐stimulation suggestive of homeostatic metaplasticity. These findings highlight the potential of tbTUS+FES as a neuromodulatory intervention, warranting further exploration in neurological conditions for therapeutic applications.

## Graphical abstract

Real theta burst transcranial focused ultrasound stimulation in combination with functional electrical stimulation resulted in immediate corticospinal inhibition followed by return to baseline suggestive of homeostatic metaplasticity. These findings highlight the potential of these combinatory techniques as a neuromodulatory intervention, warranting further exploration in neurological conditions for therapeutic applications.

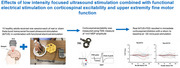

**Abbreviations**
ADM: abductor digiti minimi
BL: baseline
FDI: first dorsal interosseous
FES: functional electrical stimulation
M1: primary motor cortex
MEP: motor evoked potential
NHPT: Nine‐Hole Peg Test
NIBS: noninvasive brain stimulation
OP: opponens pollicis
PA: posterior–anterior
PAS: paired associative stimulation
tbTUS: TUS delivered in theta burst pattern
tDCS: transcranial direct current stimulation
TMS: transcranial magnetic stimulation
TUS: transcranial focused ultrasound
UHN: University Health Network

## 1 Introduction

The adult cerebral cortex is capable of undergoing functional plasticity (Nudo 1999), which can be triggered by behavioral experiences or by alterations in the nervous system (Nudo et al. 2001). In this regard, various studies have examined the effects of peripheral and central neuromodulation techniques (Chen and Udupa 2009; Ni and Chen 2015; Furlan et al. 2022) and applied this knowledge to develop treatments for neurological conditions (Furlan et al. 2022; Kesikburun 2022; Naro and Calabrò 2022; Kapadia, Nagai, et al. 2014). Most neurological conditions disrupt sensorimotor networks and cause widespread cortical activation changes leading to movement loss (Nudo 2013). While rehabilitation remains the primary treatment, restoring successful movement execution remains challenging (Nudo 2013).

Functional electrical stimulation (FES) is a neurorehabilitation technique that uses short electric impulses to generate muscle contractions in paralyzed muscles. While effective in acute stroke and incomplete spinal cord injury, its efficacy in chronic, severely affected patients is limited and variable (Kapadia, Nagai, et al. 2014; Pereira et al. 2012; Kafri and Laufer 2015; Thrasher and Popovic 2008; Jovanovic et al. 2021; Kapadia, Masani, et al. 2014; Kapadia et al. 2013, 2011; Milosevic et al. 2021). Functional performance changes following FES have been measured using functional tests like the Nine‐Hole Peg Test (NHPT) (Earhart et al. 2011; Xu et al. 2012).

More recently, noninvasive brain stimulation (NIBS) techniques such as transcranial magnetic stimulation (TMS) and transcranial direct current stimulation (tDCS) have shown efficacy as treatment (Lefaucheur et al. 2020; Lefaucheur et al. 2017). However, NIBS such as TMS lacks spatial resolution and depth of penetration. Transcranial focused ultrasound stimulation (TUS) is a novel NIBS technique that offers superior spatial resolution (2–4 mm) and the ability to target superficial and deep brain regions (Fomenko et al. 2018; Darmani et al. 2022). Focusing the propagation of acoustic waves through the skull, TUS can safely modulate brain activities (Bergmann and Hartwigsen 2021) and induce brain plasticity that persists after completion of sonication (offline effects) (Zeng et al. 2022, 2024, 2022, 2024). The neuromodulatory effects of TUS can be excitatory or inhibitory with the use of different sonication parameters (Zeng et al. 2024; Gibson et al. 2018; Zadeh et al. 2024). Our group has shown that 80 s of theta burst TUS (tbTUS) increases motor cortex (M1) excitability for at least 30 min and alters its connectivity with other brain regions, suggesting neuroplastic changes in the brain (Zeng et al. 2022; Grippe et al. 2024). These changes were blocked by calcium channel and glutamatergic *N*‐methyl‐d‐aspartate (NMDA) receptor blockers, suggesting long‐term potentiation (LTP)‐like effects (Shamli Oghli et al. 2023). In a recent study by Ding et al., the authors found that tbTUS when delivered before continuous theta burst stimulation resulted in depotentiation and abolishment of the tbTUS‐induced plasticity as measured by 1 mV motor evoked potential (MEP) amplitude (Ding et al. 2024). Whereas, when tbTUS was delivered after continuous theta burst stimulation, it resulted in metaplasticity‐type effects, causing the tbTUS‐induced plasticity to last longer (greater than 60 min) compared to tbTUS alone (Ding et al. 2024). These LTP‐like effects may have potential application toward the use of TUS as a rehabilitation modality in neurological disorders (Simonetta‐Moreau 2014; Samuel et al. 2023). Previous studies have used a combination of peripheral and central neuromodulation techniques to retrain motor function (Milosevic et al. 2021; Milosevic et al. 2020).

However, there remains limited information on how TUS interacts with other plasticity protocols. Past studies have shown that TUS‐induced plasticity can last up to 60 min post‐sonication. Given its potential to induce LTP‐like effects, combining it with techniques like FES could maximize rehabilitation outcomes.

In this proof of concept study in healthy volunteers, we aimed to assess the neuromodulatory effects of combining tbTUS and FES of intrinsic hand muscles during the execution of a fine motor task using TMS measures of corticospinal excitability. We hypothesized that combining tbTUS with FES would increase corticospinal excitability compared to FES alone and improve NHPT performance more than FES alone.

## 2 Materials and Methods

Fifteen healthy participants were recruited through advertisements at the University Health Network (UHN). The cohort included 8 females, age range 19–38 years (mean age ± standard deviation [SD] = 27.07 ± 4.71 years); 14 participants were right‐hand dominant. None of the participants were taking any medications known to affect brain excitability or had a history of neurological or psychiatric disorders. All the participants had a normal neurological examination conducted by a neurologist. The study was approved by the UHN (Toronto) Research Ethics Board. All participants provided written informed consent, and the experimental procedures were performed in accordance with the Declaration of Helsinki.

**FIGURE 1:**
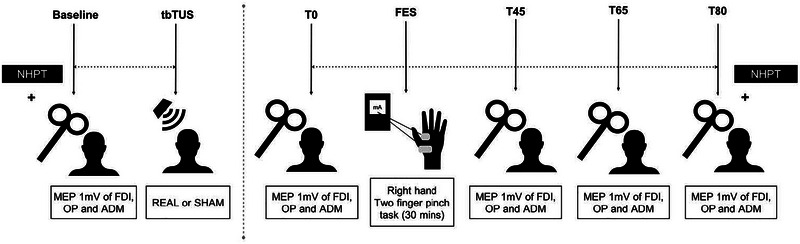
Overview of the experimental paradigm. Participants were studied in separate sessions with “real” or “sham” tbTUS followed by FES. Measure of cortical excitability (set to induce 1 mV MEP amplitude in the FDI muscle at baseline) and hand function (NHPT) were assessed for up to 80 min after the end of tbTUS. FES involved stimulation of the FDI and OP muscles during a pinch grip training task for 30 min. ADM, abductor digiti minimi; FDI, first dorsal interosseous; FES, functional electrical stimulation; MEP, motor‐evoked potential; NHPT, Nine‐Hole Peg Test; OP, opponens pollicis; T0, immediately after tbTUS; T45, immediately after FES; T65, 15 min after FES; T80, 30 min post‐FES; tbTUS, theta burst focused ultrasound.

All study participants underwent two study visits consisting of real (active condition) or sham tbTUS (sham condition) of the left M1 immediately followed by 30 min of FES training for fine motor function of the right hand (Figure 1). The order of session type (real or sham) was randomized across participants, who were blinded to the type of stimulation. The two study visits were spaced at least a week apart to allow for a washout period. During TMS recordings, participants were constantly monitored by the research personnel to ensure that they did not fall asleep.

### 2.1 tbTUS of M1

Participants were seated comfortably in a chair. tbTUS was delivered using a custom two‐element annular array ultrasound transducer (H246; Sonic Concepts, Bothell, WA) with a fundamental frequency of 0.5 MHz, a diameter of 38 mm, and a thickness of 10 mm. A programmable radiofrequency amplifier (Transducer Power Output System TPO201‐80, Sonic Concepts) delivered the required power to the transducer via a 50 Ω impedance matching module. The sonication depth was set as 30 mm according to the scalp cortex distance to the hand motor area. The acoustic beam properties using the above parameters have been modeled in a previous study from our laboratory; characterization of the ultrasound parameters was carried out in a water tank containing degassed, deionized, and distilled water (Zeng et al. 2022). This ensured that our parameters were below the recommended FDA clinical ultrasound guidelines for living tissue and significantly below the 5.9 W/cm^2^ that was applied in prior human studies with no side effects (Sarica et al. 2022). The free water values for the depth of 30 mm were 11.73 W/cm^2^ for spatial peak‐pulse‐average intensity (ISPPA) and 1.17 W/cm^2^ for spatial peak‐time average intensity (ISPTA). The estimation of the ISPPA and ISPTA at the brain target (30 mm) were 2.93 and 0.29 W/cm^2^, respectively, based on a 75% attenuation due to skull density (Mueller et al. 2017; Fomenko et al. 2020).

The motor representation (hotspot) of the right first dorsal interosseous (FDI) muscle in the left M1 was identified by TMS as the target for TUS. The hotspot was defined as the location over which the largest and most consistent MEPs were elicited by TMS with the handle of the coil pointed backward and laterally at about 45° from the midsagittal line (induced currents in the posterior–anterior [PA] direction) (Julkunen et al. 2009). The hotspot was marked on the skull with a pen to center the ultrasound transducer over the target and to ensure reliable TUS and TMS coil repositioning during the experiments. During sonication, conductive gel (Wavelength MP Blue Multi‐Purpose Ultrasound Gel, Sabel Med, Oldsmar, FL) covering the ultrasound transducer in contact with the scalp was placed onto the hotspot identified by TMS. The real tbTUS paradigm consisted of an 80‐s train of 20 ms ultrasound bursts, repeated every 200 ms (pulse repetition frequency [PRF] = 5 Hz, duty cycle = 10%, total number of bursts = 400) (Zeng et al. 2022; Samuel et al. 2022). The power of real tbTUS was set at 20 W/cm^2^. Sham tbTUS involved flipping the transducer such that the active face was directed away from the scalp and ultrasonic energy was not transmitted into the head. For both the real and sham tbTUS conditions, Gaussian white noise was generated by an internal sound card in a computer and delivered to participants through earphones to mask the auditory stimulus from the transducer. The onset and offset time of white noise was the same as tbTUS. The volume was set as high as the participants could tolerate.

### 2.2 FES

Participants were seated in a chair with a table in front of them adjusted so that they could rest their arms on the table with the shoulder in ∼20°–30° of abduction in the scapular plane and the elbow in ∼90° flexion. FES was delivered using a battery‐operated stimulator (EV‐906 Digital TENS/EMS, Everyway Medical Instruments Company) under the supervision of a researcher. The stimulation parameters were pulse width 300 µs, frequency 40 Hz, ramp time 1 s, on time 2 s, and off time 1 s. The FES session lasted for 30 min (total stimulation “ON time” was 20 min). Surface electrodes were placed over the motor points of the right FDI and opponens pollicis (OP) muscles. Pulse amplitude (range: 10–20 mA) was adjusted as per participant comfort and to elicit a functional contraction to produce a two‐finger pinch grip. During the FES task, participants were required to pick up pebbles from one dish using a two‐finger pinch and drop them into another dish. Participants were instructed to use their voluntary effort and synchronize the pick‐up phase with the “ON” time and the release phase with the “OFF” time of the stimulation. They completed 450 trials in a 30‐min session. Figure 1 shows the experimental protocol.

### 2.3 Outcome Measures

Measurement of corticospinal excitability: The primary neurophysiological outcome of interest was the TMS measurement of corticospinal excitability. Twenty MEPs were recorded at the minimum stimulus intensity required to elicit 1 mV MEP at baseline (BL) in the relaxed FDI muscle. The same stimulus intensity was used at all follow‐up assessments (T0, T45, T65, and T80).

TMS was applied to the left M1 at the FDI hotspot with a 70 mm coil figure‐of‐eight connected to 4 Magstim (Whitland, UK) 200^2^ stimulators via a “4‐to‐1” box. MEPs were recorded from the right FDI, OP, and abductor digiti minimi (ADM) muscles through pairs of 9 mm‐diameter surface Ag‐AgCl electrodes in a belly‐tendon montage. The electromyographic (EMG) signals were amplified at 1K (Model 2024F; Intronix Technologies Corporation, Bolton, ON, Canada), filtered (bandpass 20Hz–2.5 kHz), digitized at 5 kHz (Micro 1401; Cambridge Electronic Design, Cambridge, UK), and stored in a laboratory computer for offline analyses.

During each study visit, TMS‐elicited MEP data were recorded from the FDI hotspot: before any neuromodulation (BL), immediately after sonication (T0), after 30 min of FES training (T45), and at 15 (T65) and 30 min (T80) post‐FES training. When designing the study, the goal was to measure changes in MEP amplitudes 15 and 30 min post‐FES. However, in reality, when conducting the experiment, it took longer to set up and remove the FES electrodes and set up the TMS. In the manuscript, we state the actual times post‐sonication at which TMS measures were recorded, which were T45, T65, and T80. Between the time points of T45, T65, and T80, the participants were instructed to sit quietly in a chair. At all time points, the stimulus intensity determined at BL was used (active session 55.1% ± 11.6%; sham session 56.2% ± 11.7% stimulator output).

#### 2.3.1 Fine motor function assessment

Fine motor function was assessed using the NHPT, a timed assessment of manual dexterity often used to assess digit dexterity for M1 excitability studies and measurement of fine motor learning (Grover et al. 2023). Participants, while seated comfortably with their arms resting on the table at waist level, transferred nine pegs from a well into vertical holes and back using a two‐finger pinch as quickly as possible (Earhart et al. 2011). One practice run was allowed at BL for the right hand. Time taken for each hand was recorded separately at BL and T80 during both study visits.

### 2.4 Statistical Methods

Peak‐to‐peak MEP amplitudes for each trial were measured using a customized script in Signal software (Cambridge Electronic Design, Cambridge, UK) and then averaged for each condition. The ratio to MEP amplitude at BL was calculated for both real and sham conditions for four time points (T0, T45, T65, and T80), referred to as normalized MEP ratios. All MEP values reported are normalized MEP ratios. Statistical analyses were performed using IBM SPSS Version 28 (IBM Corp.). The Shapiro–Wilk test was employed to assess the normality of the data distributions for the peak‐to‐peak MEP amplitudes recorded from the FDI, OP, and ADM muscles. The test results indicated that the data were non‐normally distributed. Consequently, nonparametric statistical methods were utilized for analysis. For within‐group comparisons, the Wilcoxon signed‐rank test was used to analyze changes in peak‐to‐peak MEP amplitudes at different time points (BL, T0, T45, T65, and T80) within each condition (real and sham tbTUS). For analyzing the time × condition interaction effects, change scores were computed for each time point relative to BL for both the active and sham groups, and the Wilcoxon signed‐rank test was applied to the change scores across the two conditions. Statistical significance was set at *p* < 0.05, and Bonferroni correction was applied to adjust for multiple comparisons, ensuring the robustness of the results. The Bonferroni correction took into consideration five comparisons, and hence the adjusted *p* value of significance changed from *p* < 0.05 to *p* < 0.01.

## 3 Results

All 15 study participants completed both study visits, and no adverse events were reported. The average duration between the two study visits was 11.6 days (range: 6–30 days). MEP data from one subject was excluded from analysis due to significant background EMG activity identified with visual inspection of the raw data. Hence, data for 14 participants were analyzed. Due to data storage issues, NHPT data was lost for two participants for the sham condition and for three participants for the active condition.

The stimulus intensity that elicited MEP amplitudes of ∼1 mV for the FDI muscle at BL was comparable between the two conditions (active 55.1% ± 11.6% vs. sham 56.2% ± 11.7% stimulator output). The BL MEP amplitudes for the FDI (active 1.29 ± 0.31 mV vs. sham 1.19 ± 0.35 mV), OP (active 1.07 ± 0.60 mV vs. sham 0.97 ± 0.71 mV), and ADM muscles (active 0.85 ± 0.63 mV vs. sham 0.91 ± 0.78 mV) for real and sham conditions were also similar.

**FIGURE 2:**
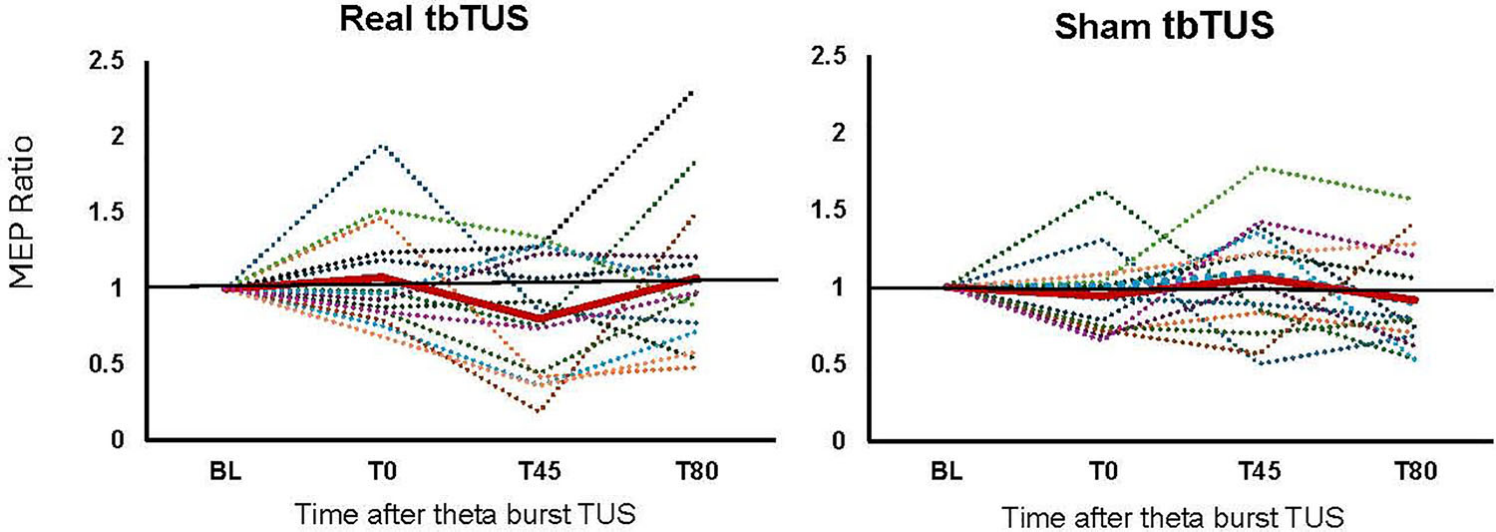
Effect of tbTUS + FES FDI MEP amplitudes in individual participants. Data are plotted as a ratio to the baseline MEP. Ratios higher than 1 indicate facilitation and ratios below 1 indicate inhibition. In the real tbTUS condition, immediately following active tbTUS + FES, MEP amplitudes decreased in the FDI muscle but this was not seen in sham tbTUS + FES condition. ADM, abductor digiti minimi; FDI, first dorsal interosseous; MEP, motor evoked potential; OP, opponens pollicis; TUS, transcranial ultrasound; T0, time post‐sonication; T45, time post‐FES; T65, 15 min post‐FES; T80, 30 min post‐FES.

The Wilcoxon signed‐rank test showed that MEP amplitudes for the FDI muscle did not change significantly from BL to T0 following either real (1.29 ± 0.31–1.31 ± 0.25 mV, *z* = −0.031, *p* = 0.97) or sham condition (1.19 ± 0.35–1.13± 0.46 mV, *z* = −1.036, *p* = 0.30). Similarly, no significant changes were seen from BL to T45 following either real tbTUS (1.29 ± 0.31–1.00 ± 0.52 mV, *z* = −1.41, *p* = 0.158) or sham tbTUS (1.19 ± 0.35–1.30 ± 0.62, *z* = −0.847, *p* = 0.40) However, MEP amplitudes for the FDI muscle significantly decreased from T0 to T45 (1.31 ± 0.25–1.00 ± 0.52 mV, *z* = −2.229, *p* = 0.03) for the active condition with no significant changes in the sham condition (1.13± 0.46–1.30 ± 0.62 mV, *z* = −1.350, *p* = 0.18). The effect size, measured by Cohen's *d*, was *d* = 0.70, indicating a medium effect. After correcting for multiple comparisons, however, this result was no longer significant (*p* > 0.01). No significant changes were seen from BL to T80 for both conditions. Figure 2 shows individual participant changes in FDI MEP amplitude ratios at different time points relative to BL.

The Wilcoxon signed‐rank test showed that MEP amplitudes for the OP muscle did not change significantly from BL to T0 following either real (1.07 ± 0.60–1.05 ± 0.64, *z* = −0.094, *p *= 0.925) or sham tbTUS (0.97 ± 0.70–0.82 ± 0.52, *z* = −0.471 *p *= 0.638). However, there was a significant decrease from BL to T45 (1.07 ± 0.60–0.79 ± 0.66 mV, *z* = −2.22, *p *= 0.026) for real tbTUS. The effect size, measured by Cohen's *d*, was *d* = 0.37, indicating a small effect. There was no significant change from BL to T45 in the sham tbTUS condition (0.97 ± 0.70–0.81 ± 0.43 mV, *z* = −0.659, *p *= 0.638). Similarly, MEP amplitudes for the OP muscle showed a significant decrease from T0 to T45 (1.05 ± 0.64–0.79 ± 0.66 mV, *z* = −2.29 *p* = 0.022) for the real tbTUS condition with no significant change in the sham tbTUS condition (0.82 ± 0.52–0.81 ± 0.43 mV, *z* = −0.094, *p* = 0.925). The effect size, measured by Cohen's *d*, was *d* = 0.33, indicating a small effect. After correcting for multiple comparisons, however, neither of these results was significant (*p* > 0.01). There were no significant changes for the OP muscle from BL to T80 for either of the two conditions.

MEP amplitudes for the ADM muscle did not show significant changes between any two time points for either of the two conditions.

A statistically significant difference was found for the FDI peak‐to‐peak 1 mV MEP scores between the active [active (T45) − active (BL)] versus sham conditions [sham (T45) − sham (BL)] (−0.291 ± 0.58 and 0.107 ± 0.39 mV, respectively) (*z* = −2.040, *p* = 0.041). No statistically significant differences were found between the APB and ADM peak‐to‐peak 1 mV MEP change scores between the active and sham conditions at any other time points.

**FIGURE 3:**
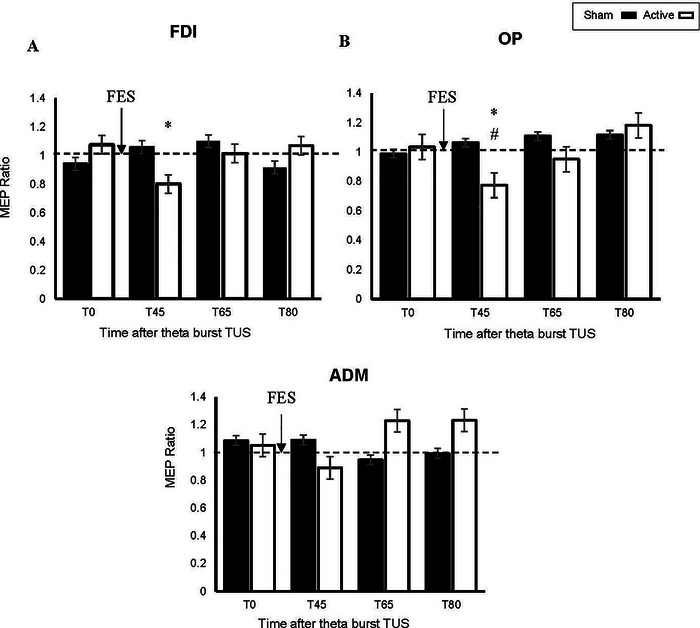
Effect of tbTUS + FES on MEP amplitudes. Data are plotted as a ratio to the baseline MEP amplitude indicated by the dashed line. Ratios higher than 1 indicate facilitation and ratios below 1 indicate inhibition. In the FDI and the OP muscles, active tbTUS + FES decreased MEP amplitudes immediately following tbTUS + FES but not in sham tbTUS + FES condition. *Significant difference using the Wilcoxon signed‐rank test between T0 and T45, *p* = 0.026 (uncorrected), ^#^Significant difference using the Wilcoxon signed‐rank test between baseline and T45, *p* = 0.022 (uncorrected). Error bars represent standard error. ADM, abductor digiti minimi; FDI, first dorsal interosseous; MEP, motor evoked potential; OP, opponens pollicis; TUS, transcranial ultrasound; T0, time post‐sonication; T45, time post‐FES; T65, 15 min post‐FES; T80, 30 min post‐FES.

Figure 3 shows the MEP ratios for the FDI, OP, and ADM muscles in the active and sham conditions.

### 3.1 NHPT

The Wilcoxon signed‐rank test showed no significant changes in the time taken to complete the NHPT with the right hand after the active or sham conditions (active BL = 18.86 ± 2.19 s; active T80 = 19.30 ± 2.60 s and sham BL = 18.60 ± 1.24 s; sham T80 = 18.75 ± 1.86 s). Similarly, no significant changes were observed in the time taken to complete the NHPT with the left hand after the active or sham conditions (active BL = 21.86 ± 2.23 s; active T80 = 20.96 ± 2.86 s and sham BL = 21.33 ± 2.11 s; sham T80 = 20.76 ± 1.68 s). There was no significant difference in the change scores of the left and right hands between the two conditions (*z* = −1.16, *p* = 0.110).

## 4 Discussion

We investigated how cortical priming using tbTUS delivered over the M1, followed by 30 min of FES training during voluntary execution of a pinch grip task, affected corticospinal excitability of hand muscles. Contrary to our hypothesis, our results revealed that tbTUS followed by FES resulted in an immediate corticospinal inhibition (T45), which was not present in the sham tbTUS condition. The MEP amplitudes returned to BL at T80 in the real tbTUS condition. Changes in MEP amplitudes may be due to changes in cortical and spinal excitability, or both. Below, we discuss the potential mechanisms underlying the changes we observed when delivering tbTUS + FES or FES alone.

### 4.1 Effect of the tbTUS Alone

The MEP did not change significantly from BL to T0 in both real or sham conditions for the FDI, OP, or ADM muscles, even though tbTUS has previously been reported to be an excitatory protocol. There are several reasons for this observation. Previous studies have shown significant inter‐individual variability in the timing of tbTUS‐related plasticity (Zeng et al. 2022; Ding et al. 2024); we also found inter‐individual variability in MEP values amongst our study participants. Besides, different studies have assessed MEP amplitudes at different time points (Zeng et al. 2022; Shamli Oghli et al. 2023; Samuel et al. 2022). One study that assessed the mechanisms of the tbTUS showed increased MEP amplitudes at 5 min, but the largest increase was observed at 30 min after tbTUS (Grippe et al. 2024; Shamli Oghli et al. 2023). Another study showed increased MEP amplitudes 15–20 min after tbTUS sonication, but earlier timepoints were not assessed (Samuel et al. 2022). In the study by Grippe et al., which used the same tbTUS protocol as the current study, the authors found a statistically significant increase in 1 mV MEP amplitudes at T30 relative to BL in the healthy control (Grippe et al. 2024). A review evaluated the plasticity effects of another NIBS protocol, intermittent theta burst (iTBS) TMS stimulation, and showed that the highest MEP amplitudes were obtained at 10 or 15 min after the stimulation compared to 5 min, suggesting that LTP‐like effects might not be detected immediately after the intervention (Wischnewski and Schutter 2015). Therefore, previous studies indicate that the timing of MEP increase after tbTUS varies between individuals; therefore, the absence of an immediate MEP increase does not mean there was no excitatory effect.

### 4.2 Real tbTUS in Combination With FES during Voluntary Movement Execution

Priming the M1 with the excitatory tbTUS protocol prior to FES resulted in an initial corticospinal inhibition after FES, which later returned to BL corticospinal excitability. Although these findings, when corrected for multiple comparisons, were not significant, 9 of the 14 study participants showed this trend in the FDI, and 12 of the 14 participants showed this trend in the OP muscle. A possible reason for decreased MEP amplitudes in the FDI and OP muscles immediately after FES (T45) is that the combination of tbTUS and FES, both excitatory protocols, when applied one after another, might have triggered homeostatic plasticity, an intrinsic regulatory mechanism, thereby reducing or reversing the effect on corticospinal excitability towards that of depression. Homeostatic plasticity is a well‐described physiological mechanism of the brain to consolidate memory and to “balance” the overall brain excitability (Lanza et al. 2022). Ding et al. showed that plasticity was abolished when an inhibitory TMS protocol (cTBS with 150 pulses) was delivered after tbTUS, indicating that plasticity induction by tbTUS can be modified in manners consistent with homeostatic metaplasticity (Ding et al. 2024). In the present study this homeostatic response at T45 suggests that the tbTUS changed cortical excitability, which may be most prominent during FES (Lanza et al. 2022).

Several studies combined different forms of cortex stimulation with peripheral stimulation of nerves or muscles to induce corticospinal plasticity (Milosevic et al. 2020). There is evidence for homeostatic regulation of paired associative stimulation (PAS)‐induced plasticity in the motor and sensory cortices (Suppa et al. 2017; Müller et al. 2007). The LTP/LTD‐like effects of the PAS protocol can be manipulated by priming with another plasticity protocol. Priming with a preceding LTP‐inducing PAS session decreased LTP induction of the subsequent PAS intervention (Müller et al. 2007). However, these changes are complex and are dependent on factors such as the time between two plasticity protocols, the level of motor activity, and there are inter‐individual differences (Müller‐Dahlhaus et al. 2015; Lepage et al. 2012; Cirillo et al. 2009; Ni et al. 2014). An excitatory PAS priming protocol suppresses subsequent motor learning in accordance with homeostatic metaplasticity (Kang et al. 2011; Elahi et al. 2014). However, the effects of this interaction are dose dependent, as a study showed that priming the M1 with an excitatory iTBS protocol followed by 10 min of FES resulted in increased MEP amplitude in both the targeted and the non‐targeted muscles, and the effect lasted for 30 min post‐FES (Cao et al. 2022). These findings were attributed to the gating mechanism, which can increase synaptic strength.

FES alone‐mediated changes in function are associated with increased corticospinal excitability as shown by increased MEP amplitudes post‐FES in clinical trials (Milosevic et al. 2020; Sasaki et al. 2017; Ridding et al. 2000; Ridding et al. 2001). Since tbTUS is also excitatory in nature and is likely related to LTP‐like mechanisms (Shamli Oghli et al. 2023), our findings are consistent with homeostatic plasticity. Another notable finding is that in our study the initial corticospinal inhibition recorded in the FDI and OP muscles reversed to BL at T80 (not observed in the ADM). This may be due to the high spatial resolution of tbTUS and the fact that the FES targeted only the FDI and OP muscles (Darmani et al. 2022).

### 4.3 Sham tbTUS Followed by FES During Voluntary Movement Execution

In our study, a 30‐min session of only FES combined with task‐oriented voluntary movement did not result in a significant increase in MEP amplitude. Corticospinal excitability following electrical stimulation is influenced by various factors, including stimulation parameters (Milosevic et al. 2020). Previous studies suggest that peripheral stimulation above motor threshold, inducing voluntary‐like contractions at higher frequencies, is needed to reliably increase corticospinal excitability (Milosevic et al. 2020).

Patients with neurological conditions have varying motor cortical BL excitability and respond differently to plasticity protocols, potentially affecting the interaction between tbTUS and FES (Leodori et al. 2022; McDonnell and Stinear 2017; Bai et al. 2022). Impaired homeostatic plasticity mechanisms may contribute to this variability (Chen et al. 2022). A randomized controlled trial showed that inhibitory low‐frequency rTMS (1 Hz) followed by FES increased motor excitability in stroke patients compared to FES alone (Yang et al. 2022), suggesting that FES effects are influenced by BL cortical excitability. This aligns with the Bienenstock–Cooper–Munro (BCM) rule, which posits that LTP induction is harder when neural activity is high (Müller et al. 2007). Further studies are needed to explore the combined effects of excitatory tbTUS and routine rehabilitation on neurophysiology and functional outcomes in patients with neurological conditions.

The current study has several limitations. First, neuronavigation was not used to locate the M1 hotspot, which might have increased MEP variability, although the M1 representation of the FDI muscle can still be reliably identified by consistent MEP amplitudes (Jung et al. 2010). How neuronavigation affects the targeting and neuromodulatory effects of TUS of M1 requires further study. Moreover, we did not perform individual modeling of the effects of TUS, and the acoustic pressure at the target may have varied from subject to subject, which might have increased the variability of the results. Second, MEP amplitudes were not measured beyond the T80 time point, limiting our ability to observe long‐term effects of tbTUS+FES. Third, the effects of tbTUS alone were not measured, though previous studies have documented the excitatory nature of tbTUS in healthy individuals (Fomenko et al. 2018; Darmani et al. 2022; Zeng et al. 2022, 2024, 2022, 2024; Shamli Oghli et al. 2023; Sarica et al. 2022; Fomenko et al. 2020; Samuel et al. 2022). Fourth, our small sample size may limit the generalizability of our findings, suggesting a need for larger studies to confirm these results and explore further implications. Moreover, we did not inquire whether participants noticed any differences between the real and sham TUS conditions; it is possible that some individuals were able to discern the stimulation they received. Finally, to thoroughly explore the combined effect of tbTUS and FES, the condition of real tbTUS + sham FES should have been included.

## 5 Conclusion

This study demonstrated that the combination of low‐intensity TUS (tbTUS) and FES can modulate corticospinal excitability. Specifically, the pairing of tbTUS with FES resulted in an initial decrease in MEP amplitudes, followed by a return to BL levels, suggesting a homeostatic metaplastic response. Based on our results, we propose that tbTUS has the potential to influence the neuroplastic effects of FES, offering a promising avenue for augmenting motor function retraining during rehabilitation. However, these results need to be replicated in a larger sample. Future research needs to explore the synergistic effects of tbTUS and FES and optimize protocols for use in rehabilitation of motor function.

## Author Contributions

**Naaz Desai**: conceptualization, methodology, data curation, investigation, formal analysis, project administration, writing–review and editing, writing–original draft. **Talyta Grippe**: data curation, writing–review and editing, methodology. **Tarun Arora**: methodology, writing–review and editing, investigation. **Amitabh Bhattacharya**: methodology, writing–review and editing, visualization. **Carolyn Gunraj**: project administration, resources, writing–review and editing. **Robert Chen**: conceptualization, methodology, funding acquisition, supervision, visualization, writing–review and editing.

## Ethics Statement

Before data collection, ethics approval was obtained from the University Health Network (Toronto) Research Ethics Board.

## Consent

All participants signed an informed consent form before participation in the research study.

## Conflicts of Interest

The authors declare no conflicts of interest.

### Peer Review

The peer review history for this article is available at https://publons.com/publon/10.1002/brb3.70318.

## Data Availability

The data that support the findings of this study are available from the corresponding author upon reasonable request.

## References

[brb370318-bib-0001] Bai, Z. , J. Zhang , and K. N. K. Fong . 2022. “Effects of Transcranial Magnetic Stimulation in Modulating Cortical Excitability in Patients With Stroke: A Systematic Review and Meta‐Analysis.” Journal of Neuroengineering and Rehabilitation 19, no. 1: 24. 35193624 10.1186/s12984-022-00999-4 PMC8862292

[brb370318-bib-0002] Bergmann, T. O. , and G. Hartwigsen . 2021. “Inferring Causality From Noninvasive Brain Stimulation in Cognitive Neuroscience.” Journal of Cognitive Neuroscience 33, no. 2: 195–225. 32530381 10.1162/jocn_a_01591

[brb370318-bib-0003] Cao, N. , A. Sasaki , A. Yuasa , M. R. Popovic , M. Milosevic , and K. Nakazawa . 2022. “Effective Corticospinal Excitability Neuromodulation Elicited by Short‐Duration Concurrent and Synchronized Associative Cortical and Neuromuscular Stimulations.” Neuroscience Letters 790: 136910. 36228774 10.1016/j.neulet.2022.136910

[brb370318-bib-0004] Chen, L. , X. Li , M. Tjia , and S. Thapliyal . 2022. “Homeostatic Plasticity and Excitation‐Inhibition Balance: The Good, the Bad, and the Ugly.” Current Opinion in Neurobiology 75: 102553. 35594578 10.1016/j.conb.2022.102553 PMC9477500

[brb370318-bib-0005] Chen, R. , and K. Udupa . 2009. “Measurement and Modulation of Plasticity of the Motor System in Humans Using Transcranial Magnetic Stimulation.” Motor Control 13, no. 4: 442–453. 20014649 10.1123/mcj.13.4.442

[brb370318-bib-0006] Cirillo, J. , A. P. Lavender , M. C. Ridding , and J. G. Semmler . 2009. “Motor Cortex Plasticity Induced by Paired Associative Stimulation Is Enhanced in Physically Active Individuals.” Journal of Physiology 587, no. Pt 24: 5831–5842. 19858227 10.1113/jphysiol.2009.181834 PMC2808543

[brb370318-bib-0007] Darmani, G. , T. O. Bergmann , K. Butts Pauly , et al. 2022. “Non‐Invasive Transcranial Ultrasound Stimulation for Neuromodulation.” Clinical Neurophysiology 135: 51–73. 35033772 10.1016/j.clinph.2021.12.010

[brb370318-bib-0008] Ding, M. Y. R. , T. Arora , C. Sarica , et al. 2024. “Investigation of Metaplasticity Associated With Transcranial Focused Ultrasound Neuromodulation in Humans.” Journal of Neuroscience 44, no. 44: e2438232024. 39266303 10.1523/JNEUROSCI.2438-23.2024 PMC11529810

[brb370318-bib-0009] Earhart, G. M. , J. T. Cavanaugh , T. Ellis , M. P. Ford , K. B. Foreman , and L. Dibble . 2011. “The 9‐Hole PEG Test of Upper Extremity Function: Average Values, Test‐Retest Reliability, and Factors Contributing to Performance in People With Parkinson Disease.” Journal of Neurologic Physical Therapy 35, no. 4: 157–163. 22020457 10.1097/NPT.0b013e318235da08

[brb370318-bib-0010] Elahi, B. , W. D. Hutchison , Z. J. Daskalakis , C. Gunraj , and R. Chen . 2014. “Dose‐Response Curve of Associative Plasticity in Human Motor Cortex and Interactions With Motor Practice.” Journal of Neurophysiology 111, no. 3: 594–601. 24198319 10.1152/jn.00920.2012

[brb370318-bib-0011] Fomenko, A. , K. S. Chen , J. F. Nankoo , et al. 2020. “Systematic Examination of Low‐Intensity Ultrasound Parameters on Human Motor Cortex Excitability and Behavior.” eLife 9: e54497. 33236981 10.7554/eLife.54497 PMC7728443

[brb370318-bib-0012] Fomenko, A. , C. Neudorfer , R. F. Dallapiazza , S. K. Kalia , and A. M. Lozano . 2018. “Low‐Intensity Ultrasound Neuromodulation: An Overview of Mechanisms and Emerging Human Applications.” Brain Stimulation 11, no. 6: 1209–1217. 30166265 10.1016/j.brs.2018.08.013

[brb370318-bib-0013] Furlan, J. C. , M. Pakosh , B. C. Craven , and M. R. Popovic . 2022. “Insights on the Potential Mechanisms of Action of Functional Electrical Stimulation Therapy in Combination with Task‐Specific Training: A Scoping Review.” Neuromodulation: Technology at the Neural Interface 25, no. 8: 1280–1288. 34031937 10.1111/ner.13403

[brb370318-bib-0014] Gibson, B. C. , J. L. Sanguinetti , B. W. Badran , et al. 2018. “Increased Excitability Induced in the Primary Motor Cortex by Transcranial Ultrasound Stimulation.” Frontiers in Neurology 9: 1007. 30546342 10.3389/fneur.2018.01007 PMC6280333

[brb370318-bib-0015] Grippe, T. , Y. Shamli‐Oghli , G. Darmani , et al. 2024. “Plasticity‐Induced Effects of Theta Burst Transcranial Ultrasound Stimulation in Parkinson's Disease.” Movement Disorders 39, no. 8: 1364–1374. 38787806 10.1002/mds.29836

[brb370318-bib-0016] Grover, F. M. , B. Chen , and M. A. Perez . 2023. “Increased Paired Stimuli Enhance Corticospinal‐Motoneuronal Plasticity in Humans with Spinal Cord Injury.” Journal of Neurophysiology 129, no. 6: 1414–1422. 36752493 10.1152/jn.00499.2022 PMC10259851

[brb370318-bib-0017] Jovanovic, L. I. , N. Kapadia , V. Zivanovic , et al. 2021. “Brain‐Computer Interface‐Triggered Functional Electrical Stimulation Therapy for Rehabilitation of Reaching and Grasping After Spinal Cord Injury: A Feasibility Study.” Spinal Cord Series and Cases 7, no. 1: 24. 33741900 10.1038/s41394-020-00380-4 PMC7979732

[brb370318-bib-0018] Julkunen, P. , L. Säisänen , N. Danner , et al. 2009. “Comparison of Navigated and Non‐Navigated Transcranial Magnetic Stimulation for Motor Cortex Mapping, Motor Threshold and Motor Evoked Potentials.” NeuroImage 44, no. 3: 790–795. 18976714 10.1016/j.neuroimage.2008.09.040

[brb370318-bib-0019] Jung, N. H. , I. Delvendahl , N. G. Kuhnke , D. Hauschke , S. Stolle , and V. Mall . 2010. “Navigated Transcranial Magnetic Stimulation Does Not Decrease the Variability of Motor‐Evoked Potentials.” Brain Stimulation 3, no. 2: 87–94. 20633437 10.1016/j.brs.2009.10.003

[brb370318-bib-0020] Kafri, M. , and Y. Laufer . 2015. “Therapeutic Effects of Functional Electrical Stimulation on Gait in Individuals Post‐Stroke.” Annals of Biomedical Engineering 43, no. 2: 451–466. 25316590 10.1007/s10439-014-1148-8

[brb370318-bib-0021] Kang, J. S. , C. Terranova , R. Hilker , A. Quartarone , and U. Ziemann . 2011. “Deficient Homeostatic Regulation of Practice‐Dependent Plasticity in Writer's Cramp.” Cerebral Cortex 21, no. 5: 1203–1212. 20974689 10.1093/cercor/bhq204

[brb370318-bib-0022] Kapadia, N. , K. Masani , B. Catharine Craven , et al. 2014. “A Randomized Trial of Functional Electrical Stimulation for Walking in Incomplete Spinal Cord Injury: Effects on Walking Competency.” Journal of Spinal Cord Medicine 37, no. 5: 511–524. 25229735 10.1179/2045772314Y.0000000263 PMC4166186

[brb370318-bib-0023] Kapadia, N. , V. Zivanovic , and M. R. Popovic . 2013. “Restoring Voluntary Grasping Function in Individuals With Incomplete Chronic Spinal Cord Injury: Pilot Study.” Topics in Spinal Cord Injury Rehabilitation 19, no. 4: 279–287. 24244093 10.1310/sci1904-279 PMC3816722

[brb370318-bib-0024] Kapadia, N. M. , M. K. Nagai , V. Zivanovic , et al. 2014. “Functional Electrical Stimulation Therapy for Recovery of Reaching and Grasping in Severe Chronic Pediatric Stroke Patients.” Journal of Child Neurology 29, no. 4: 493–499. 23584687 10.1177/0883073813484088

[brb370318-bib-0025] Kapadia, N. M. , V. Zivanovic , J. C. Furlan , B. C. Craven , C. McGillivray , and M. R. Popovic . 2011. “Functional Electrical Stimulation Therapy for Grasping in Traumatic Incomplete Spinal Cord Injury: Randomized Control Trial.” Artificial Organs 35, no. 3: 212–216. 21401662 10.1111/j.1525-1594.2011.01216.x

[brb370318-bib-0026] Kesikburun, S. 2022. “Non‐Invasive Brain Stimulation in Rehabilitation.” Turkish Journal of Physical Medicine and Rehabilitation 68, no. 1: 1–8. 35949977 10.5606/tftrd.2022.10608 PMC9305642

[brb370318-bib-0027] Lanza, G. , L. M. DelRosso , and R. Ferri . 2022. “Sleep and Homeostatic Control of Plasticity.” In Handbook of Clinical Neurology, edited by A. Quartarone , M. F. Ghilardi , and F. Boller , 53–72. Elsevier. 10.1016/B978-0-12-819410-2.00004-7 35034758

[brb370318-bib-0028] Lefaucheur, J. P. , A. Aleman , C. Baeken , et al. 2020. “Evidence‐Based Guidelines on the Therapeutic Use of Repetitive Transcranial Magnetic Stimulation (rTMS): An Update (2014–2018).” Clinical Neurophysiology 131, no. 2: 474–528. 31901449 10.1016/j.clinph.2019.11.002

[brb370318-bib-0029] Lefaucheur, J. P. , A. Antal , S. S. Ayache , et al. 2017. “Evidence‐Based Guidelines on the Therapeutic Use of Transcranial Direct Current Stimulation (tDCS).” Clinical Neurophysiology 128, no. 1: 56–92. 27866120 10.1016/j.clinph.2016.10.087

[brb370318-bib-0030] Leodori, G. , M. I. De Bartolo , A. Guerra , et al. 2022. “Motor Cortical Network Excitability in Parkinson's Disease.” Movement Disorders 37, no. 4: 734–744. 35001420 10.1002/mds.28914

[brb370318-bib-0031] Lepage, J. F. , O. Morin‐Moncet , V. Beaulé , L. de Beaumont , F. Champoux , and H. Théoret . 2012. “Occlusion of LTP‐Like Plasticity in Human Primary Motor Cortex by Action Observation.” PLoS One 7, no. 6: e38754. 22701704 10.1371/journal.pone.0038754 PMC3368919

[brb370318-bib-0032] McDonnell, M. N. , and C. M. Stinear . 2017. “TMS Measures of Motor Cortex Function After Stroke: A Meta‐Analysis.” Brain Stimulation 10, no. 4: 721–734. 28385535 10.1016/j.brs.2017.03.008

[brb370318-bib-0033] Milosevic, M. , C. Marquez‐Chin , K. Masani , et al. 2020. “Why Brain‐Controlled Neuroprosthetics Matter: Mechanisms Underlying Electrical Stimulation of Muscles and Nerves in Rehabilitation.” Biomedical Engineering Online 19, no. 1: 81. 33148270 10.1186/s12938-020-00824-w PMC7641791

[brb370318-bib-0034] Milosevic, M. , T. Nakanishi , A. Sasaki , et al. 2021. “Cortical Re‐Organization after Traumatic Brain Injury Elicited Using Functional Electrical Stimulation Therapy: A Case Report.” Frontiers in Neuroscience 15: 693861. 34489624 10.3389/fnins.2021.693861 PMC8417438

[brb370318-bib-0035] Mueller, J. K. , L. Ai , P. Bansal , and W. Legon . 2017. “Numerical Evaluation of the Skull for Human Neuromodulation With Transcranial Focused Ultrasound.” Journal of Neural Engineering 14, no. 6: 066012. 28777075 10.1088/1741-2552/aa843e

[brb370318-bib-0036] Müller, J. F. , Y. Orekhov , Y. Liu , and U. Ziemann . 2007. “Homeostatic Plasticity in Human Motor Cortex Demonstrated by Two Consecutive Sessions of Paired Associative Stimulation.” European Journal of Neuroscience 25, no. 11: 3461–3468. 17553015 10.1111/j.1460-9568.2007.05603.x

[brb370318-bib-0037] Müller‐Dahlhaus, F. , C. Lücke , M. K. Lu , et al. 2015. “Augmenting LTP‐Like Plasticity in Human Motor Cortex by Spaced Paired Associative Stimulation.” PLoS One 10, no. 6: e0131020. 26110758 10.1371/journal.pone.0131020 PMC4482149

[brb370318-bib-0038] Naro, A. , and R. S. Calabrò . 2022. “Improving Upper Limb and Gait Rehabilitation Outcomes in Post‐Stroke Patients: A Scoping Review on the Additional Effects of Non‐Invasive Brain Stimulation When Combined With Robot‐Aided Rehabilitation.” Brain Sciences 12, no. 11: 1511. 36358437 10.3390/brainsci12111511 PMC9688385

[brb370318-bib-0039] Ni, Z. , and R. Chen . 2015. “Transcranial Magnetic Stimulation to Understand Pathophysiology and as Potential Treatment for Neurodegenerative Diseases.” Translational Neurodegeneration 4: 22. 26579223 10.1186/s40035-015-0045-x PMC4647804

[brb370318-bib-0040] Ni, Z. , C. Gunraj , P. Kailey , R. F. Cash , and R. Chen . 2014. “Heterosynaptic Modulation of Motor Cortical Plasticity in Human.” Journal of Neuroscience 34, no. 21: 7314–7321. 24849363 10.1523/JNEUROSCI.4714-13.2014 PMC6608185

[brb370318-bib-0041] Nudo, R. J. 1999. “Recovery After Damage to Motor Cortical Areas.” Current Opinion in Neurobiology 9, no. 6: 740–747. 10607636 10.1016/s0959-4388(99)00027-6

[brb370318-bib-0042] Nudo, R. J. 2013. “Recovery After Brain Injury: Mechanisms and Principles.” Frontiers in Human Neuroscience 7: 887. 24399951 10.3389/fnhum.2013.00887 PMC3870954

[brb370318-bib-0043] Nudo, R. J. , E. J. Plautz , and S. B. Frost . 2001. “Role of Adaptive Plasticity in Recovery of Function After Damage to Motor Cortex.” Muscle & Nerve 24, no. 8: 1000–1019. 11439375 10.1002/mus.1104

[brb370318-bib-0044] Pereira, S. , S. Mehta , A. McIntyre , L. Lobo , and R. W. Teasell . 2012. “Functional Electrical Stimulation for Improving Gait in Persons With Chronic Stroke.” Topics in Stroke Rehabilitation 19, no. 6: 491–498. 23192714 10.1310/tsr1906-491

[brb370318-bib-0045] Ridding, M. C. , B. Brouwer , T. S. Miles , J. B. Pitcher , and P. D. Thompson . 2000. “Changes in Muscle Responses to Stimulation of the Motor Cortex Induced by Peripheral Nerve Stimulation in Human Subjects.” Experimental Brain Research 131, no. 1: 135–143. 10759179 10.1007/s002219900269

[brb370318-bib-0046] Ridding, M. C. , D. R. McKay , P. D. Thompson , and T. S. Miles . 2001. “Changes in Corticomotor Representations Induced by Prolonged Peripheral Nerve Stimulation in Humans.” Clinical Neurophysiology 112, no. 8: 1461–1469. 11459686 10.1016/s1388-2457(01)00592-2

[brb370318-bib-0047] Samuel, N. , M. Y. R. Ding , C. Sarica , et al. 2023. “Accelerated Transcranial Ultrasound Neuromodulation in Parkinson's Disease: A Pilot Study.” Movement Disorders 38, no. 12: 2209–2216. 37811802 10.1002/mds.29622

[brb370318-bib-0048] Samuel, N. , K. Zeng , I. E. Harmsen , et al. 2022. “Multi‐Modal Investigation of Transcranial Ultrasound‐Induced Neuroplasticity of the Human Motor Cortex.” Brain Stimulation 15, no. 6: 1337–1347. 36228977 10.1016/j.brs.2022.10.001

[brb370318-bib-0049] Sarica, C. , J. F. Nankoo , A. Fomenko , et al. 2022. “Human Studies of Transcranial Ultrasound Neuromodulation: A Systematic Review of Effectiveness and Safety.” Brain Stimulation 15, no. 3: 737–746. 35533835 10.1016/j.brs.2022.05.002

[brb370318-bib-0050] Sasaki, R. , S. Kotan , M. Nakagawa , et al. 2017. “Presence and Absence of Muscle Contraction Elicited by Peripheral Nerve Electrical Stimulation Differentially Modulate Primary Motor Cortex Excitability.” Frontiers in Human Neuroscience 11: 146. 28392766 10.3389/fnhum.2017.00146 PMC5364169

[brb370318-bib-0051] Shamli Oghli, Y. , T. Grippe , T. Arora , T. Hoque , G. Darmani , and R. Chen . 2023. “Mechanisms of Theta Burst Transcranial Ultrasound Induced Plasticity in the Human Motor Cortex.” Brain Stimulation 16, no. 4: 1135–1143. 37524296 10.1016/j.brs.2023.07.056

[brb370318-bib-0052] Simonetta‐Moreau, M. 2014. “Non‐Invasive Brain Stimulation (NIBS) and Motor Recovery After Stroke.” Annals of Physical and Rehabilitation Medicine 57, no. 8: 530–542. 25193774 10.1016/j.rehab.2014.08.003

[brb370318-bib-0053] Suppa, A. , A. Quartarone , H. Siebner , et al. 2017. “The Associative Brain at Work: Evidence From Paired Associative Stimulation Studies in Humans.” Clinical Neurophysiology 128, no. 11: 2140–2164. 28938144 10.1016/j.clinph.2017.08.003

[brb370318-bib-0054] Thrasher, T. A. , and M. R. Popovic . 2008. “Functional Electrical Stimulation of Walking: Function, Exercise and Rehabilitation.” Annales de Réadaptation et de Médecine Physique 51, no. 6: 452–460. 18602712 10.1016/j.annrmp.2008.05.006

[brb370318-bib-0055] Wischnewski, M. , and D. J. Schutter . 2015. “Efficacy and Time Course of Theta Burst Stimulation in Healthy Humans.” Brain Stimulation 8, no. 4: 685–692. 26014214 10.1016/j.brs.2015.03.004

[brb370318-bib-0056] Xu, K. , L. Wang , J. Mai , and L. He . 2012. “Efficacy of Constraint‐Induced Movement Therapy and Electrical Stimulation on Hand Function of Children With Hemiplegic Cerebral Palsy: A Controlled Clinical Trial.” Disability and Rehabilitation 34, no. 4: 337–346. 21961441 10.3109/09638288.2011.607213

[brb370318-bib-0057] Yang, Z. , L. Qiao , J. He , X. Zhao , and M. Zhang . 2022. “Effects of Repetitive Transcranial Magnetic Stimulation Combined With Functional Electrical Stimulation on Hand Function of Stroke: A Randomized Controlled Trial.” Neurorehabilitation 51, no. 2: 283–289. 35723120 10.3233/NRE-220074

[brb370318-bib-0058] Zadeh, A. K. , H. Raghuram , S. Shrestha , et al. 2024. “The Effect of Transcranial Ultrasound Pulse Repetition Frequency on Sustained Inhibition in the Human Primary Motor Cortex: A Double‐Blind, Sham‐Controlled Study.” Brain Stimulation 17, no. 2: 476–484. 38621645 10.1016/j.brs.2024.04.005

[brb370318-bib-0059] Zeng, K. , G. Darmani , A. Fomenko , et al. 2022. “Induction of Human Motor Cortex Plasticity by Theta Burst Transcranial Ultrasound Stimulation.” Annals of Neurology 91, no. 2: 238–252. 34964172 10.1002/ana.26294

[brb370318-bib-0060] Zeng, K. , Z. Li , X. Xia , et al. 2024. “Effects of Different Sonication Parameters of Theta Burst Transcranial Ultrasound Stimulation on Human Motor Cortex.” Brain Stimulation 17, no. 2: 258–268. 38442800 10.1016/j.brs.2024.03.001

